# Haloperidol Decanoate-Induced Pancytopenia: A Case Report

**DOI:** 10.7759/cureus.47402

**Published:** 2023-10-20

**Authors:** Amit Jagtiani, Sandra Veigne, Raghu Gandhi, Abid Rizvi

**Affiliations:** 1 Psychiatry, Burrell Behavioral Health, Springfield, USA; 2 Psychiatry, Temple University, Philadelphia, USA; 3 Psychiatry, Abbott Northwestern Hospital, Minneapolis, USA; 4 Psychiatry and Behavioral Sciences, West Virginia University, Morgantown, USA

**Keywords:** schizophrenia, pancytopenia, butyrophenone, antipsychotic, haloperidol decanoate

## Abstract

Blood dyscrasias, including pancytopenia, can rarely occur as adverse effects of antipsychotic drug therapy. While neutropenia is more common, pancytopenia remains an infrequent but serious hematological complication. We present the case of an 85-year-old African-American female with a history of schizophrenia, stabilized on haloperidol decanoate, who developed pancytopenia during her outpatient care. Her blood counts progressively declined, leading to hospitalization. Hematology evaluation ruled out infectious or neoplastic causes, implicating haloperidol decanoate-induced pancytopenia. The pancytopenia improved gradually over three months after discontinuing haloperidol decanoate. Our case highlights the importance of monitoring and timely intervention in such cases. We discuss the rarity of pancytopenia with antipsychotics and the potential mechanisms and challenging management of this condition.

## Introduction

Hematological abnormalities, known as blood dyscrasias, can manifest as a consequence of antipsychotic drug administration, potentially arising from either direct drug-induced toxicity or immunologic mechanisms leading to bone marrow suppression [1]. Among the blood dyscrasias associated with antipsychotic use, neutropenia is the most frequently observed, while thrombocytopenia occurs less frequently. Pancytopenia induced by psychotropic medications is an exceptionally rare adverse event, with predominant documentation concerning clozapine [2,3]. Nonetheless, isolated reports have also sporadically linked pancytopenia to atypical antipsychotics such as olanzapine, risperidone, and quetiapine [4-6], as well as, albeit more infrequently, with typical antipsychotics like perphenazine (a phenothiazine) [7]. The occurrence of pancytopenia with butyrophenone derivatives like haloperidol is notably less common [1]. In this article, we present a case study involving an elderly patient who developed pancytopenia while receiving monthly injections of haloperidol decanoate.

This article was previously presented as a poster at the American Association for Geriatric Psychiatry (AAGP) annual meeting in Atlanta, Georgia, United States, from March 1 to 4, 2019.

## Case presentation

We present the case of an 85-year-old African-American female with a longstanding history of schizophrenia, previously stabilized on a monthly intramuscular dose of haloperidol decanoate at 50 mg for at least five to six years during her care at another medical facility. Following her transition to outpatient care at our hospital, she continued receiving haloperidol decanoate for six months along with her antihypertensive medication amlodipine. During her initial presentation at our clinic, she was found to have pancytopenia, with a gradual decline in red blood cells (RBC), platelets, and white blood cells (WBC), including neutrophils. Consequently, her haloperidol decanoate treatment was temporarily suspended to allow for further monitoring of her blood counts.

When she initiated outpatient treatment at our clinic, her baseline WBC count was 3000/mm³, her absolute neutrophil count (ANC) was 1300/mm^3^, her hemoglobin levels were 10.9 g/dL, and her platelet count was 154,000/mm³. Over the next six months, as she continued receiving haloperidol decanoate, her leukocyte count plummeted to 1860/mm³, her ANC declined to 440/mm^3^, her hemoglobin levels fell to 10.1 g/dL, and her platelet count declined to 140,000/mm³ (Table 1). She had been on haloperidol decanoate for several years, and no information was available on her blood counts prior to its initiation. Hematology was consulted which suspected potential haloperidol decanoate-induced pancytopenia. Hence, haloperidol decanoate was discontinued. She was offered other oral antipsychotics like risperidone or aripiprazole, but she declined stating that the medications were harmful to her.

**Table 1:** Complete blood count trend during clinical care WBC: white blood cell; RBC: red blood cell; Hgb: hemoglobin; PLT: platelet K = x1000

	At baseline (at clinic intake)	At six months (haloperidol decanoate stopped)	At seven months (at hospital admission)	At hospital discharge	Three months post discharge
WBC count (K/µL)	3.0	1.86	2.1	5.1	5.6
RBC count (K/µL)	3.95	3.70	3.69	4.01	4.2
Hgb count (g/dL)	10.9	10.1	10.5	11.2	12.0
PLT count (K/µL)	154	140	105	138	151
Neutrophil count (K/µL)	1.3	0.44	0.9	2.8	3.2
Eosinophil count (K/µL)	0.0	0.0	0.0	0.0	0.0
Basophil count (K/µL)	0.0	0.0	0.0	0.0	0.0
Lymphocyte count (K/µL)	1.4	1.22	1.0	1.3	0.8
Monocyte count (K/µL)	0.2	0.19	0.2	0.5	0.1

Approximately one month after discontinuing haloperidol decanoate, the patient's clinical condition deteriorated, leading to her admission to the psychiatry inpatient unit with worsening paranoia and deterioration of self-care. Hematology consultation was again sought, and laboratory investigations aimed at excluding infectious, inflammatory, and neoplastic etiologies yielded unremarkable results.

Despite multiple efforts to administer oral medications, the patient consistently declined and eventually progressed to a catatonic state characterized by mutism, stupor, staring, and withdrawal, as indicated by a Bush-Francis catatonia rating score of 5. A court order for involuntary medication was obtained, yet the patient continued to refuse oral medications. Consequently, a consistent intramuscular dose of 5 mg of olanzapine was administered for one week before her discharge, resulting in significant clinical improvement, reduced paranoia, improved oral intake, and improved self-care. Following a two-month inpatient stay, the patient was discharged to her daughter's care, prescribed with a daily regimen of 5 mg of oral olanzapine. Throughout her hospitalization, her blood cell counts exhibited a gradual improvement. At the time of discharge, her WBC had increased to 5100/mm³, her ANC had risen to 2800/mm³, her hemoglobin levels had increased to 11.2 g/dL, and her platelet count remained stable at 138,000/mm³ (Table 1). Approximately three months after discontinuing haloperidol decanoate, her blood counts had returned to normal levels, and she continued to maintain clinical stability, free from paranoia and with satisfactory oral intake.

## Discussion

Stübner et al. conducted an extensive analysis of 122,562 patients across 35 psychiatric institutions spanning from 1993 to 2000, focusing on the induction of blood dyscrasias by psychotropic drugs [1]. Their study documented 107 cases of hematological alterations, with an incidence rate of 0.0873%. Among these cases, only two exhibited pancytopenia, while the majority presented with neutropenia (63 cases), thrombocytopenia (16 cases), agranulocytosis (22 cases), and neutropenia-thrombocytopenia (four cases).

Within the spectrum of antipsychotic medications, clozapine stands out as the agent most frequently associated with blood dyscrasias, occasionally leading to agranulocytosis, although instances of pancytopenia have also been reported [2,3]. The precise mechanisms underpinning blood dyscrasias in response to antipsychotics remain elusive, with hypotheses encompassing bone marrow suppression as a result of direct drug toxicity or immunological pathways [1]. Genetic factors, including potential associations with HLA haplotypes such as DQB*0502 and DQB1*06, have also been proposed as contributing elements [1].

Haloperidol decanoate, upon administration, undergoes gradual release into the bloodstream, undergoing immediate hydrolysis to form active haloperidol. Peak plasma concentrations are typically observed between three and nine days, and the drug exhibits an elimination half-life of approximately three weeks. Steady-state levels are generally reached after the third injection or approximately three months, equivalent to four to five times the apparent half-life [8]. Theoretically, complete elimination from the body following discontinuation would necessitate an additional three months. Consequently, idiosyncratic side effects such as blood dyscrasias may require an extended recovery period, as was observed in our patient.

The cornerstone of effective pancytopenia management revolves around early detection and prompt medication discontinuation, aimed at averting complications such as opportunistic infections and hemorrhagic events. Given that the highest-risk period is typically within the initial months of treatment, rigorous monitoring during this phase can prove beneficial. While treatments involving lithium and granulocyte colony-stimulating factor have shown promise in cases of agranulocytosis primarily associated with clozapine [9], their efficacy in cases of pancytopenia stemming from other antipsychotics remains uncertain.

## Conclusions

Blood dyscrasias should be suspected in patients treated with antipsychotics. Patients on long-acting depot antipsychotics may require a longer recovery time from blood dyscrasias due to the slow washout of depot preparations.
